# Hidden Charge‐Order in the Mixed‐Valent K_0.75_Li_2_Cr_6_O_12_ High‐Pressure Oxide

**DOI:** 10.1002/advs.202518064

**Published:** 2025-12-05

**Authors:** Angel M. Arévalo‐López, Clemens Ritter, Marielle Huvé, Olivier Mentré

**Affiliations:** ^1^ UMR‐8181‐UCCS‐Unité de Catalyse et Chimie du Solide (UCCS) CNRS Université de Lille Centrale Lille/ENSCL Lille 59000 France; ^2^ Institut Laue‐Langevin 71 Avenue des Martyrs Cedex Grenoble 32042 France

**Keywords:** charge‐order, high‐pressure oxide, magnetism, mixed‐valency, neutron diffraction

## Abstract

The mixed valent high‐pressure oxide K₀.₇_5_Li_2_Cr₆^3.54^+O_12_ is synthesized at 12 GPa and 1373 K. Synchrotron X‐ray and powder neutron diffraction (PND) reveal a *P6_3/m_* average structure. Analogous to hollandites, CrO_6_ octahedra form a corner‐sharing double‐chain framework, this creates two types of channels respectively occupied by Li^+^ and K^+^. However, electron microscopy and pair distribution function analysis suggest the loss of correlation in and between the K^+^ partially occupied channels. A magnetostrictive paramagnetic‐to‐antiferromagnetic transition occurs at *T*_N_ = 75 K, where low temperature PND reveals a propagation vector *k* = [⅓ ⅓ ¼] realising a commensurate helical structure with 45° rotation between Cr^3^⁺ and Cr⁴⁺ spins in zig‐zag ladders, stabilized by competing ferromagnetic and antiferromagnetic exchanges. Density functional theory calculations highlight the critical role of the *c*‐axis compression in determining the magnetic direct exchange interactions thus revealing the ferromagentic‐correlated paramagnet → charge‐ordered antiferromagnetic transition, reminiscent to the pressure induced FM to AFM transition in the related K_2_Cr_8_O_16_ hollandite. Despite hidden long‐range charge order due to K⁺ disorder, K₀.₇_5_Li_2_Cr₆^3.54^+O_12_ exhibits strong spin‐lattice coupling, where a slight change in the structure has a huge impact in the properties.

## Graphical abstract

K_0.75_Li_2_Cr_6_O_12_ is one of the few mixed valent chromium oxides. It shows a single angle spiral magnetic structure with a k = [1/3 1/3 ¼] propagation vector. Density functional theory reveals a hidden charge order that is necessary to stabilize the antiferromagnetic (AFM) ground state. This hidden order is induced by the inherent K^+^ positional disorder due to the loose character in its channel. The strong c compression helps on stabilizing direct‐exchange AFM interactions.

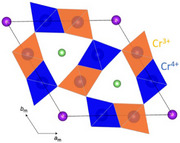

## 1 Introduction

Transition metal compounds containing mixed valent cations are a very fertile environment for interesting and useful properties. Already when considering just 3d transition metal oxides, these include famous metal‐insulator‐transitions (MIT) in the Magnéli phases of Ti and V;^[^ ^1^, ^2^, ^3^ ^]^ colossal magneto resistance (CMR) in the manganites;^[^ ^4^ ^]^ the Verwey transition along with the formation of trimerons in Fe_3_O_4_;^[^ ^5^, ^6^ ^]^ enhanced thermoelectric properties in cobaltites^[^ ^7^ ^]^ and superconductivity in nickelates and cuprates.^[^ ^8^ ^]^ However, mixed‐valent chromium oxides are scarce due to the intrinsic stability of Cr^3+^ in octahedral coordination that tends not to coexist with Cr^4+^, which prefers a tetrahedral environment. Nevertheless, some examples showing both Cr^3+^/Cr^4+^ mixing do occur; these are mainly stabilized via high‐pressure high‐temperature synthesis (HPHT). For instance, NaCr^+3.5^_2_O_4_, with the CaFe_2_O_4_‐type structure, develops an antiferromagnetic ordering along with unconventional CMR. This arises from the field spin‐reorientation that ultimately allows a better electron hopping‐path culminating in a −100% magnetoresistance at low temperature.^[^ ^9^ ^]^ Another example is the hollandite‐type K_2_Cr^+3.75^_8_O_16_, with metallic conductivity and ferromagnetic order at 180 K. At 95 K, it shows a metal‐to‐insulator transition and therefore represents a rare case of a ferromagnetic insulator.^[^ ^10^ ^]^ Moreover, this hollandite also presents a strong spin‐lattice coupling as it was demonstrated via a FM to AFM transition at 2.1 GPa, along with the possibility of a quantum critical point at higher pressure that will suppress any magnetic order even at 0 K.^[^ ^11^ ^]^ These few examples of interesting properties on mixed valence Cr‐compounds open up useful perspectives but require original candidates for generalization.

We report here the discovery of a rare mixed‐valent chromium oxide K_0.75_Li_2_Cr^3.54+^_6_O_12_ stabilized via a HPHT route. Its open framework, built on double rutile‐like chains, hosts K^+^ positional disorder that lacks perfect registry within the 3D structure. Such disorder, combined with the pristine compression along the stacking direction, indirectly induces local charge‐order hidden in the average structure. Together with spin frustration, it results in a single‐angle spiral ordering of the chromium spins, as proved by experimental and first‐principles calculations.

## 2 Results and Discussion

### 2.1 Crystal Structure

**Figure 1:**
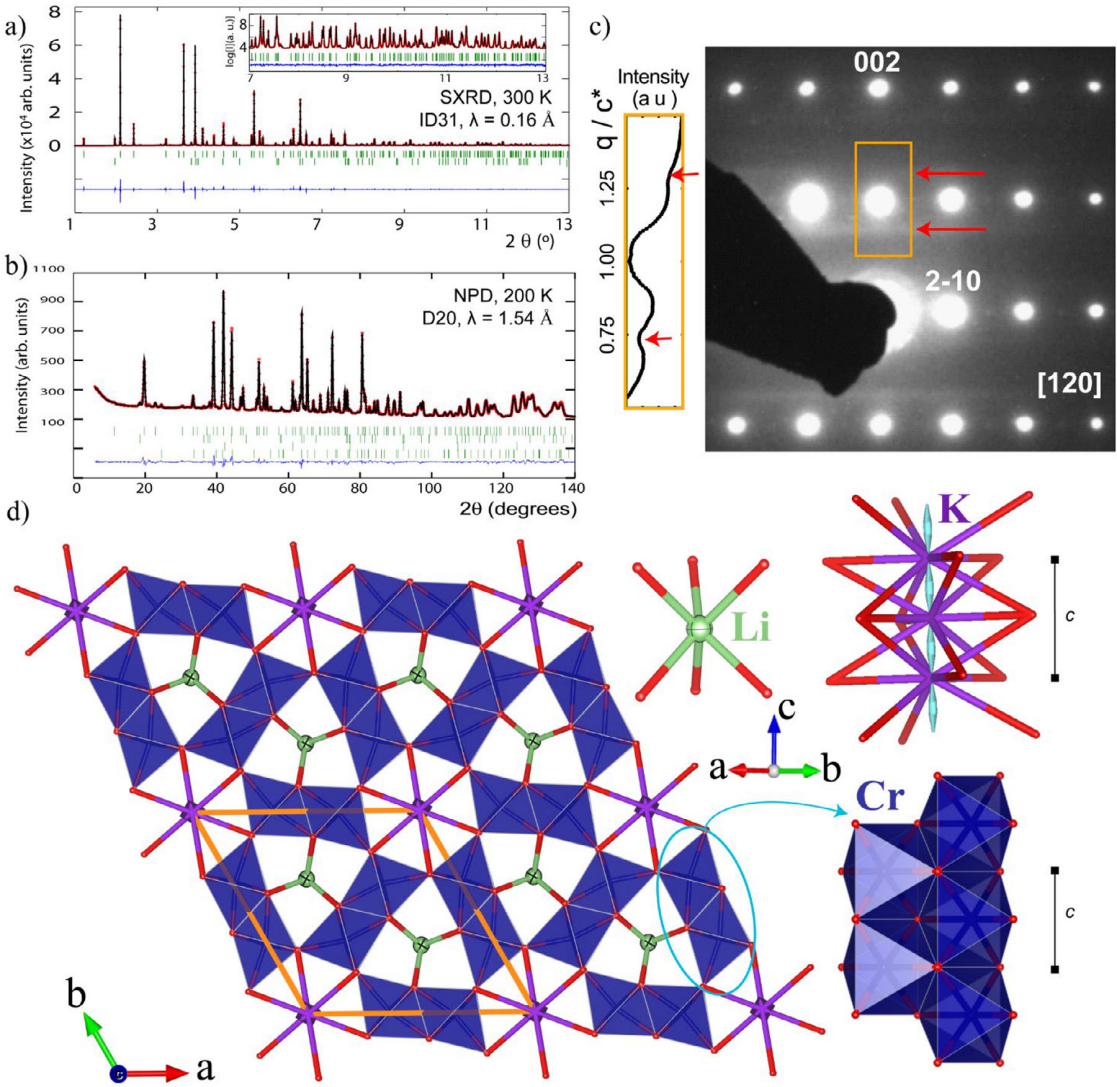
Structure of K_0.75_Li_2_Cr_6_O_12_. a) Rietveld refinement of SXRD at 300 K (λ = 0.165 Å) and b) PND at 200 K (λ = 1.54 Å). c) [120] electron diffraction along with its corresponding intensity scan of the orange square area around (001) reflection and red arrows highlighting diffuse scattering. d) K_0.75_Li_2_Cr_6_O_12_ crystal structure view along the c axis. Emphasis on CrO_6_ double‐octahedral chain, LiO_6_ triangular prism, and K^+^ hexagonal channel with disordered position in the *P6_3/m_* symmetry. Bond Valence Energy Landscape map with an iso‐energy surface of 0.56 eV in pale blue. Green, purple, and red spheres represent Li, K, and O, respectively.

K_0.75_Li_2_Cr_6_O_12_ was obtained at 12 GPa and 1373 K in a hydraulic 1000‐ton press (Societe Savoisienne de Verins Hydrauliques) using a multi‐anvil Walker‐type module (Voggenreiter), see Supporting Information (SI) for more details. The crystal structure of this new high‐pressure K_0.75_Li_2_Cr_6_O_12_ oxide was refined using synchrotron X‐ray (300 K) and neutron (200 K) powder diffraction profiles, see **Figure** **1**a,b. It crystallizes in the SrCa_2_Sc_6_O_12_ type structure^[^ ^12^ ^]^ in the *P6_3_*/*m* space group with *a* = 9.010982(2) Å and *c* = 2.86296(1) Å cell parameters at 300 K. It consists of double rutile‐chains of CrO_6_ edge‐shared octahedra running along the *c*‐direction interconnected by corners in the *ab*‐plane and thus forming an open framework with two types of channels as shown in Figure 1d. The Li^+^ cations reside inside the small channel in a triangular prism coordination. The larger hexagonal channels formed by six corner‐sharing octahedra are occupied by large K^+^ cations.

The refinement of the anisotropic thermal ellipsoids of the K^+^ atoms resulted in large values along the *c*‐axis, for example, *U*_33_ = 0.195(12) Å^3^. These unusually strong anisotropic thermal ellipsoids have also been reported in, for instance, Pb_0.78_Li_2_Rh_6_O_12_ or KLi_2_Ru_6_O_12_.^[^ ^13^, ^14^ ^]^ Fourier‐difference maps suggest 2a and 2b positions [(0, 0, ¼) and (0, 0, 0)] with correct KO_x_ coordination, respectively, to be simultaneously partially occupied (occ. ≤ 1/4, i.e. maximum one K^+^ per unit cell). This implies positional disorder of K^+^ along the *z* direction, that is, inside each channel with a loss of correlation between adjacent channels. Figure 1d shows the cigar‐shaped bond valence energy landscapes (BVEL) calculations for K^+^ at 0.05 eV percolation energy,^[^ ^15^ ^]^ thus suggesting a possible K^+^ diffusion path along the channel.

At the microscopic level, the situation is quite different. More than 15 crystals were tested via Electron Dispersive X‐ray (EDX) spectroscopy and resulted in a ≈0.25 K^+^ deficiency with a 1:8 K: Cr ratio after calibration with K_2_Cr_2_O_7_, see Supporting Information. Figure 1c shows the electron diffraction (ED) along the [120] direction. It exposes the existence of the (001) reflection, not originating from kinematic double diffraction effects, as it is preserved during sample‐tilting. At the range of the ED coherence‐length, this is consistent with the local breaking of the 6_3_ axis, thus the loss of the K^+^ translation by ½ *c* (≈1.4 Å). The ED pattern also exhibits diffuse scattering streaks (cuts to diffuse sheets) perpendicular to the *c* direction and at ≈±¼ of the main reflections. In structurally related hollandite compounds, similar diffuse scattering has been related to Ba/Ti partial ordering in the tunnels of Ba_x_M_8‐__y_Ti_y_O_16_ (M = Zn, Co, Mg, Fe, and Mn).^[^ ^16^, ^17^ ^]^ In the same way, in Ba_0.85_Ca_−2.15_In_6_O_12_, an incommensurately modulated order between Ba and Ca along the channels with a *q* = (1/3a*, 1/3b*, 0.1385c*) modulation vector has been observed.^[^ ^18^ ^]^ Therefore, these diffuse sheets imply short‐range order of the interstitial K^+^/vacancies (*Vc*) along their channel direction but with little or no transverse correlation from channel to channel. Note that in the bulk, the (001) reflection was not observed in the SXRD or the PND data, and the *P6_3_*/*m* symmetry was maintained with the K^+^ disorder. We then allowed to refine the K^+^ occupancy and its position in the synchrotron data, these converged to 0.73(1) occupied and z ≈ 0.137, almost in the middle of 0 and ¼ from the special positions. We leave it fixed to 0.75 in accordance with EDX analysis for the neutron refinement but change it to the 4e position along with the reduced occupancy, allowed to refine K^+^ thermal factors isotropically to *Biso*_K_ = 0.78(24) Å^3^ at 200 K. Li^+^ occupancy was also allowed to vary but refined to nominal values and was kept fixed, still with Li^+^ *U*_33_ = 0.040(4) Å^3^, which is observed in for instance in LiFeAs_2_O_7_ or LiFeO_2_,^[^ ^19^, ^20^ ^]^ see Supplementary Information. However, BVEL calculations for Li^+^ did not show any possibility for percolation. Moreover, BVS calculations show that Li^+^ coordination is almost ideal and may reflect a contraction effect highlighted by DFT as discussed later. Therefore, the average model with *P6_3_*/*m* symmetry shows partial K^+^ occupancy (75%) and positional disorder within and between its channels. Nonetheless, this K^+^ deficiency has a minor impact on the Cr average oxidation state of K_0.75_Li_2_Cr_6_O_12_, changing it from its ideal +3.5 to +3.54, calculated to +3.62(8) at 200 K (PND), ICSD deposit No. 2504388.

**Figure 2:**
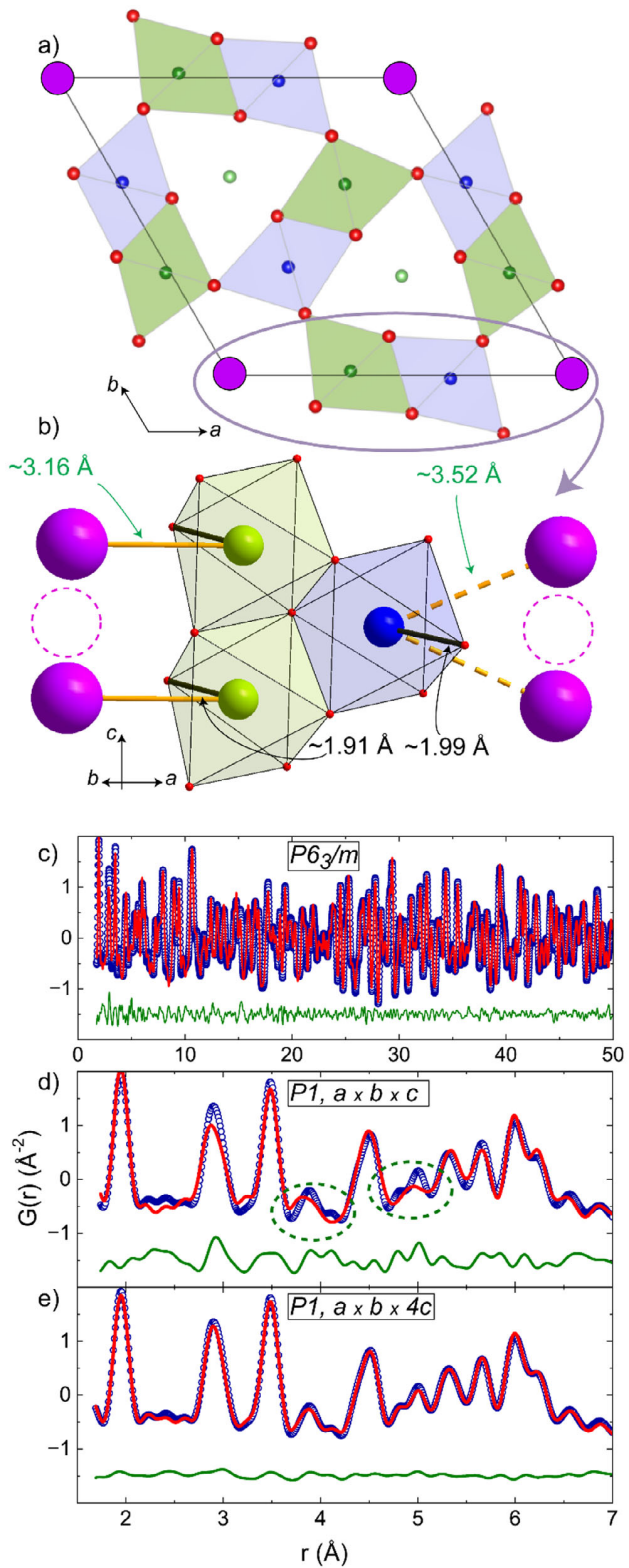
a) DFT+U relaxed single cell structure, which resulted in *P‐6* symmetry with two distinctive CrO_6_ octahedra with a long/short (1.99/1.91 Å) Cr–O distance toward K atoms, as shown in b) vacancies are also shown as broken circles. c) PDF result at long distances with the average structure *P6_3_*/*m*. d) PDF removing all the symmetry constraints in a single cell at short distances. e) PDF in a 4*c* supercell allowing K^+^ occupancy and positions to refine along with the coordinated oxygens.

However, this K^+^ deficiency clearly plays a role, as demonstrated via DFT + U calculations. Starting with a single cell and with K^+^ atoms half‐occupied in the 2a site of the channels [K in (0,0,0) and vacancy in (0,0,½)], the relaxed structure segregates two different Cr–O_3_ distances (1.91/1.99 Å), see **Figure** **2**a,b. Accordingly, the Cr sites are split into two positions due to this K^+^/Vc ordering and thus formally corresponds to a lowering of the crystal symmetry from *P6_3_*/*m* to *P‐6*. Subsequently, we have also relaxed a 2*a* x *b* x 4*c* supercell, starting from two types of 75% occupied channels, with partially occupied 2a or 2b K^+^ and *Vc* starting positions. After relaxation, the distribution of the K^+^ atoms along the channels is such that, regardless of the initial configuration, see Figure S2 (Supporting Information), they become equidistant along the channel‐axis with significant but stable shifts from their original positions. This supports the idea of easy K^+^‐ion diffusion in the channels as observed from the BVEL, see SI for details.

In order to qualitatively probe the influence of the K^+^/*Vc* correlated disorder on local deviations from the average structure in the bulk, we performed X‐ray pair distribution function analysis (PDF). Experimental PDFs for K_0.75_Li_2_Cr_6_O_12_ are shown in Figure 2c–e. The least‐squares‐refined PDF, solid red line in the Figure, was obtained using the SXRD Rietveld refined structure as input for PDFGUI and provides an excellent fit to the large‐r data (Figure 2c), but at shorter distances, the experimental PDF is poorly modelled. Even the removal of any symmetry constraints cannot fit the PDF properly at the local level. The largest mismatches at ≈4 and ≈5 Å describe only single peaks where there are clear doublets, see Figure 2d. In order to incorporate the microscopy observations, we used a fourfold supercell along *c* into the fit. This supercell was allowed to refine the K^+^ occupancy and their displacements along the channels. In order to resemble our DFT calculations, the oxygen atoms involved in the K^+^ coordination were also allowed to refine (*P1* symmetry). As shown in Figure 2e, an excellent fit is obtained, with four 75% occupied K^+^ (0, 0, z) positions and a distribution of Cr–O distances (from 1.79 to 2.07 Å) which is broader than using the ideal *P6_3_*/*m* model (1.93–1.97 Å). However, we did not observe abnormally high thermal parameters for oxygen positions, at least using powder diffraction. The supercell PDF model also suggests a tendency to columnar charge‐ordering (CO), but at very short correlation lengths, see Supporting Information. At the end, in K_0.75_Li_2_Cr_6_O_12_, the bulk crystallography averages all these effects. Nevertheless, it is remarkable that the K^+^ inter‐ and intra‐channel disorder with low‐energy diffusion and the concerned oxygen positions may act as premises for hidden charge‐ordering, as discussed later.

### 2.2 Properties

**Figure 3:**
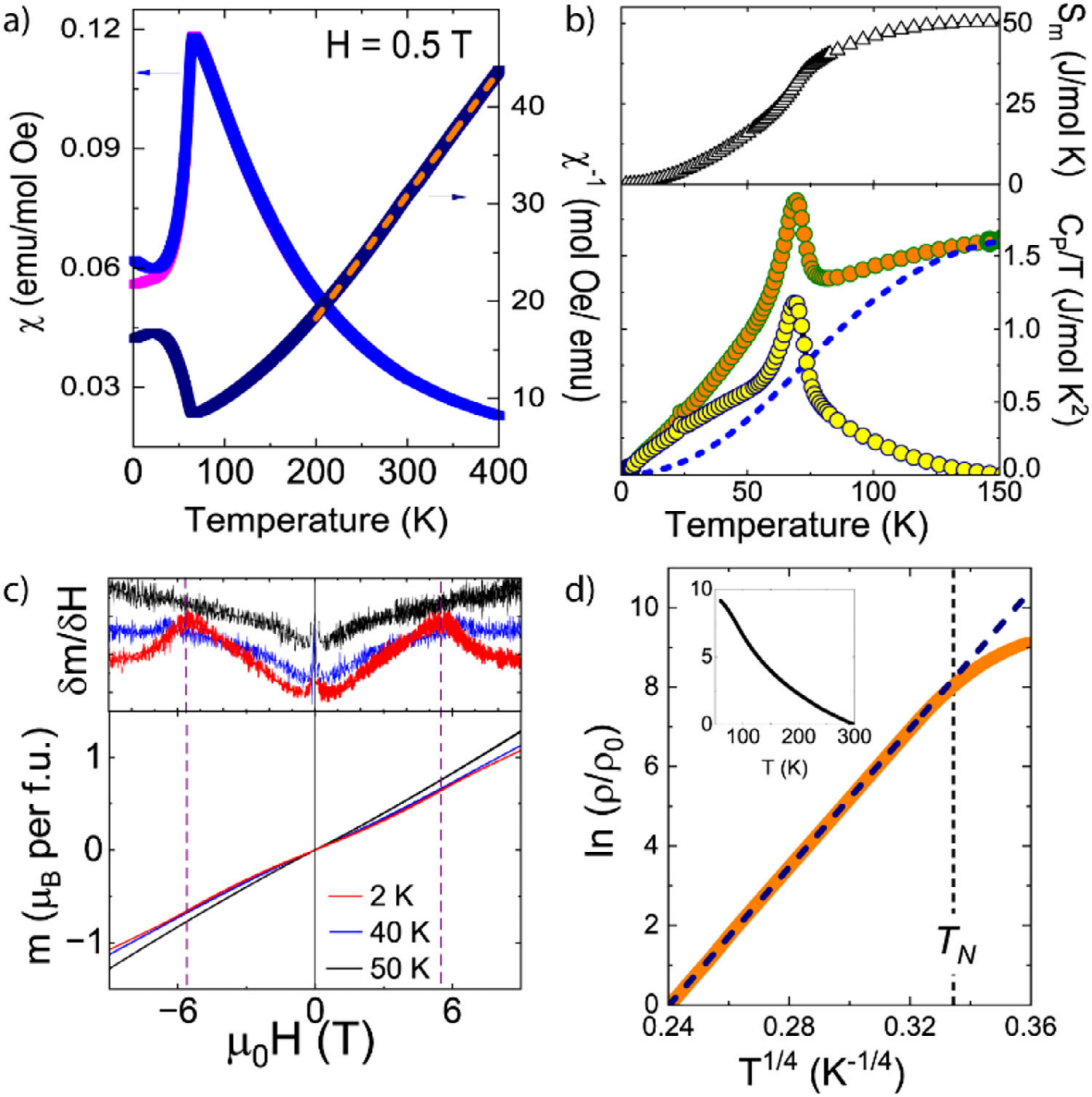
K_0.75_Li_2_Cr_6_O_12_ properties. a) Direct and inverse magnetic susceptibility measured at 0.5 T external field. The Curie–Weiss law fitted above 200 K is shown as a broken line. b) Specific heat divided by temperature (orange dots) along with magnetic (yellow dots) and lattice contributions (dashed blue line) (bottom). Calculated magnetic entropy (top). c) Field‐dependent magnetization at 2, 40, and 50 K (bottom). The derivative shows a sudden change at ± 5.4 T only for 2 and 40 K (top). d) Variation of ln(ρ/ρ0) versus T^−1/4^, the inset shows ln(ρ/ρ_0_) as a function of temperature. The broken dashed line represents the fit to the Variable Range Hopping model, see text for details.

The thermal evolution of the magnetic susceptibility χ for K_0.75_Li_2_Cr_6_O_12_ is depicted in **Figure** **3**a. It reveals a Curie–Weiss paramagnetic behavior in the 200–400 K temperature range, with Weiss temperature of *θ*_CW_ = 70 K demonstrating predominant ferromagnetic interactions and an effective moment of *µ*_eff_ = 3.24(2) µ_B_/Cr close to the localized spin‐only value *µ*_theo_ = 3.35 µ_B_/Cr for a 0.46:0.54 Cr^3+^:Cr^4+^ ratio. However, only a small divergence between field‐cooled and zero‐field‐cooled measurements at low temperatures is observed, and no hysteresis is detected in the field dependence magnetization at 2 K, as shown in Figure 3c, thus revealing an antiferromagnetic behaviour. Moreover, a field‐induced weak spin reorientation is observed below 40 K and ≈ 6 T, as observed in the derivative as an upper inset in Figure 3c. The shape of the hysteresis could be due to domain reorientation, sample texture effects, or changes in the spin structure;^[^ ^21^ ^]^ due to the AFM nature of the curve, the first two options were excluded. Additional heat capacity measurements in Figure 3b also show a transition at *T*_N_ = 75 K, associated with the onset of the 3D long‐range antiferromagnetic ordering. The recovered magnetic entropy reaches its saturation at ≈ 50 J/mol K at 150 K, it represents ≈ 80% of the expected configurational spin entropy ΔS = R (3ln4 + 3ln3) = 62 J/mol K for 3 x Cr^3+^ (S = 3/2) and 3 x Cr^4+^ (S = 1).

The temperature dependence of the electrical resistivity (ρ) of a dense K_0.75_Li_2_Cr_6_O_12_ polycrystalline pellet as obtained from HPHT synthesis at zero magnetic field is shown in the inset of Figure 3d. Resistivity measurements under 9 T did not show any significant magnetoresistance, contrary to NaCr_2_O_4_, where unconventional colossal magnetoresistance was reported.^[^ ^9^ ^]^ This is probably attributed to the different exchange interactions along the zig‐zag chains, which are FM in the latter but AFM in K_0.75_Li_2_Cr_6_O_12_ as discussed below. The ρ(T) increases with decreasing temperature, implying semiconductivity and displaying an inflexion around *T*_N_ toward more localized electronic states. Below 60 K, ρ(T) goes beyond our measurement limit. Attempts to fit the data to the Arrhenius law showed linear behaviour only at high temperature (200–300 K) with a small activation energy of 0.17(2) eV (see Supporting Information). A much better fit is obtained with Mott's 3D variable range hopping model (VRH) with ρ(T) = ρ_0_ [exp(T_0_/T)^1/4^], where it only deviates from linearity below *T*_N_ as shown in Figure 3d.

### 2.3 Magnetic Structure

To better understand the magnetic behaviour of K_0.75_Li_2_Cr_6_O_12_, powder neutron diffraction (PND) data at lower temperatures were collected on the D20 diffractometer at the Institut Laue Langevin, Grenoble. Additional Bragg reflections were observed below ≈ 78 K with a sudden increase in intensity on cooling. The reflections were consistent with *k*_1_ = [⅓ ⅓ ¼] and *k*_2_ = [0 0 ¼] (see Supporting Information) and followed the same intensity‐temperature dependence. However, further analysis via ISOSUBGROUP shows that *k*_2_ is a second‐order parameter of *k*_1_ and therefore a better description emerges via the magnetic space group in order to include the symmetry allowed spin degrees of freedom, as nicely exemplified in Tb_14_Ag_51_ and Ho_2_BaCuO_5_.^[^ ^22^, ^23^ ^]^

**Model 1 (No CO)**: The second best fit to the 10–100 K difference data was obtained in the 1.3 *P*_S_1 magnetic space group with a {(210)(‐110)(004)} magnetic supercell (R_mag_ = 7.7%, χ^2^ = 2.4). It is a result of mP2P3, mP1 and mΔ4Δ6 irreps acting together in the chromium sites. Cr moments lie in the *ab* plane and were initially constrained with an equal moment; they refined to 1.93(1) µ*B*, which is almost fully saturated for Cr^4+^ (S = 1) but lacking one ordered spin for Cr^3+^ (S = 3/2). Although we cannot rule out the possibility of similar magnetic structures giving equally good fits to the data, the imposed constraint to have the same moment for each chromium could be expected from considering *k*_2_ = 3*k*_1_ as the appearance of a third‐harmonic. The presence of odd‐order harmonics generally results in the squaring‐up of an amplitude‐modulated spin structure, for instance, in HoPdAl_4_Ge_2_.^[^ ^24^ ^]^ **Model 2 (CO)**: However, as discovered by DFT + U calculations described below, we have constrained the magnetic moments in the large magnetic supercell to two types of chromium in each of the double‐rutile chains ordered per column, the moments refine to 1.37(4) and 2.88(4) µ_B_ for Cr^4+^ (blue/cyan) and Cr^3+^ (red/orange), respectively, resulting in a clear improvement of the model (R_mag_ = 4.1%, χ^2^ = 2.16). Ideally, at the crystallographic level, such segregation by column corresponds to the symmetry lowering from *P6_3_*/*m* to *P‐6* already mentioned above, which allows two different chromium sites. However, one needs to keep in mind that neutron diffraction is only sensitive to components of the magnetisation perpendicular to the scattering vector. Since the main magnetic reflection is (001) in the supercell, this implies that the most relevant interaction is related to the ordering between planes along *c*, which is the helical structure discussed below.

**Figure 4:**
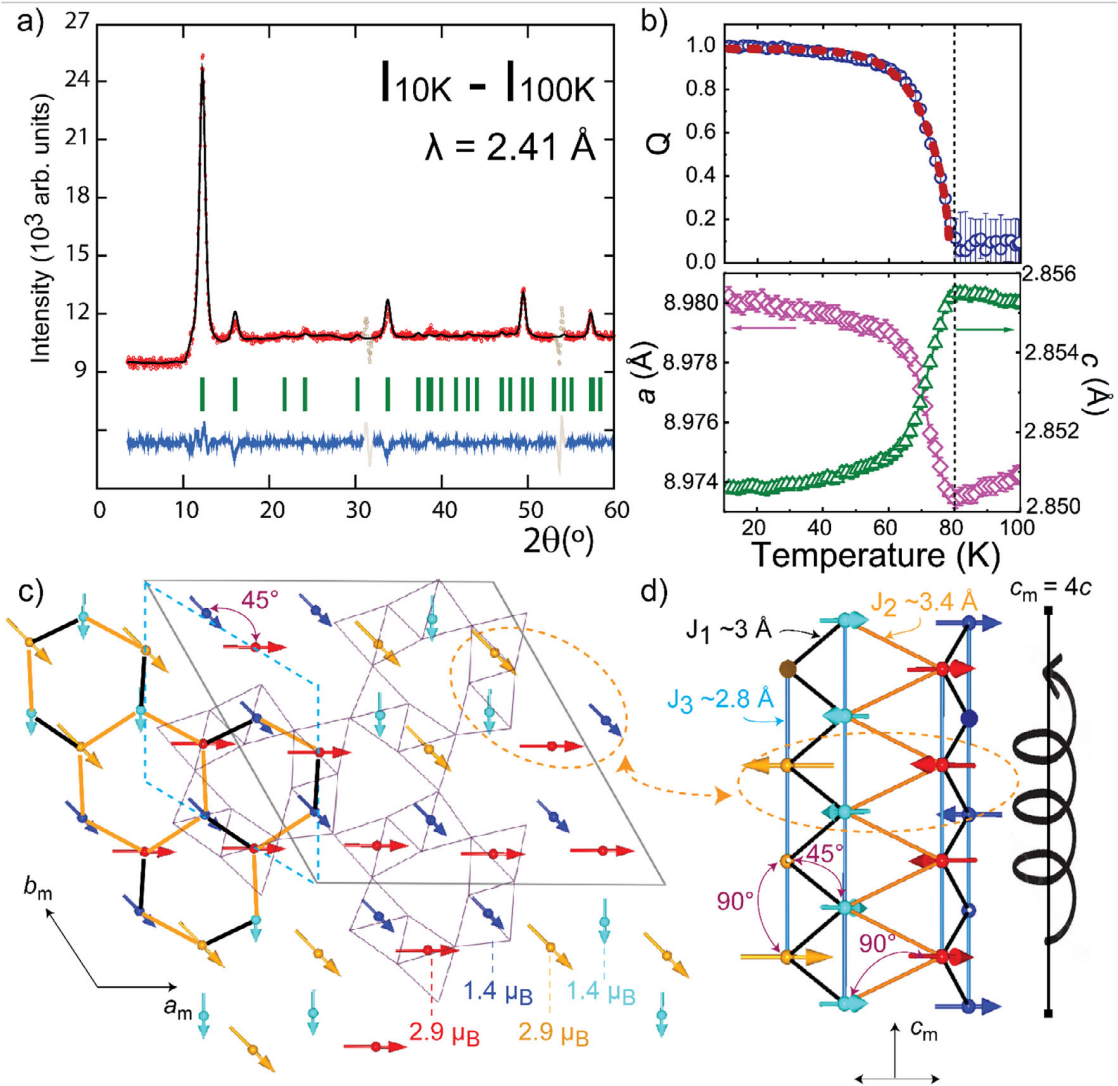
Magnetic structure of K_0.75_Li_2_Cr_6_O_12_. a) Rietveld refinement of the Intensity difference between 10 and 100 K PND data with the P1.1′c [BNS 1.3 P_S_1] magnetic space group. b) Chromium magnetic moment order parameter (Q) and fit to the function described in the text to Q (top). *a* and *c* cell parameters thermal evolution from PND data. Error bars are smaller than the points (bottom). c) Magnetic structure of K_0.75_i_2_Cr_6_O_12_ at 10 K projected on the *xy* plane and with only ¼ of *c*_m_ shown. The crystal unit cell is indicated by the dotted blue line. The black solid line shows the √3a x √3b magnetic supercell. Red(orange) and blue(cyan) spins are rotated 45° indicated by an arrow. d) Two double chains are shown with their respective spins rotated ±45° and 90° in between the chains as marked by arrows; each zig‐zag chain can be understood as a 45° spiral ordering.

The refinement profile and magnetic structure are shown in **Figure** **4**. The latter can be described on the basis of a zig‐zag triangular ladder topology, where each ladder is formed by the Cr^3+^–Cr^4+^ double‐rutile chains as shown in Figure 4d, similar to β‐CaCr_2_O_4_.^[^ ^25^ ^]^ In between every node of the rung, there is a 45° spin rotation. The connection between different double chains is either via a ±45° or 0/90° spin rotation (red‐blue or blue‐orange/red‐cyan, respectively, in the figure). It is this “out‐of‐phase” that gives the √3 x √3 character to the magnetic structure.

The thermal evolutions of the magnetic moment and the cell parameters are shown in Figure 4b. Cooling through *T*_N_, K_0.75_Li_2_Cr_6_O_12_ displays magnetostriction in line with the anisotropic cell dimensions, exhibiting an expansion in the *ab* plane and a contraction along the *c* axis. Likewise, the magnetic moments increase their values up to 1.37(4) and 2.88(4) µ_B_ at 10 K. Attempts to adjust a simple critical law to the thermal evolution of the moments were unsuccessful. Nonetheless, we use the empirical function Q_A_ = tanh(*W*t^β^)/tanh(*W*), where t = (*T*_c_–*T*)/*T*_c_ for temperatures *T* in K and with *W* acting as fitting parameters, this equation allows for higher order contributions to the Landau free energy expansion at large *t*, in agreement with the mixing of the magnetic irreps.^[^ ^26^, ^27^ ^]^ Initial refinement gave β = 0.52(3), consistent with β = ½ from mean field theory, and the critical exponent was fixed at the latter value, resulting in *T*_c_ = 77(1) K.

### 2.4 Calculations

In order to understand the magnetic structure of K_0.75_Li_2_Cr_6_O_12_, the spin exchange interactions were inspected via DFT + U (U = 3 eV) calculations. To keep the number of exchanges meaningful, three different interactions were considered. They are referred to as *J*_1_ ~ 3 Å, *J*_2_ ~ 3.4 Å, and *J*_3_ ~ 2.8 Å for edge‐sharing (intra‐double chain), corner‐sharing (inter‐double chain), and direct exchange (stringer of the ladder), respectively, see Figure 4d. Relaxing the structure using different (super)cells (6 or 12 Cr/cell) with ferromagnetically polarized spins always resulted to equivalent subcells with *a*_FM_ = 8.98 Å and *c*_FM_ = 2.89 Å, which when compared to the experimental values at 20 K *a*_20K_ = 8.9801(5) Å and *c*_20K_ = 2.8505(5) Å, displayed a clearly expanded *c*_FM_ parameter. Moreover, the obtained Cr magnetic moment values were calculated between 2.52 and 2.61 μ_B_/Cr, implying a mixed valence state. The three exchange interactions were determined using the method described in Ref. [28], leading to only FM exchanges (−218, −201, and −16 K for *J*_1,2,3_/kB, respectively). Different U values of 1.5 and 5 eV were also computed, but all the interactions remained FM without complying with the experimental AFM observations.

On the contrary, after relaxation with an initial simplified model (12 Cr/supercell) mimicking the experimental magnetic structure, that is, FM in the layer but AFM coupled along z (*J*_3_) as shown in Supporting Information, the cell parameters converged to the equivalent subcell with *a*_AFM_ = 9.02 Å and *c*_AFM_ = 2.87 Å, closer to *c*_20K_ and with a 2.8 and 2.2 μ_B_/Cr charge ordering (CO) segregation in the double‐rutile chains, see Supporting Information. The exchange interactions for this model, averaged from several local values due to the Cr^3+^/Cr^4+^ CO, resulted in FM *J*_1/kB_ = −107.5 K and *J*_2/kB_ = −154.7 K but AFM J_3/kB_ = 108.5 K.

Globally, the AFM model suggests the ideal *P6_3_*/*m* symmetry is broken and that CO occurs at *T*_N_. The model is also in accordance with the magnetoelastic behavior of K_0.75_LiCr_6_O_12_ shown in Figure 4b, where, by analogy, the transition from Cr^3.54^+ paramagnetic with FM correlations → Cr^3+^/Cr^4+^ CO AFM shows an expansion/contraction of the *a*/*c* cell parameters, see Supporting Information.

**Figure 5:**
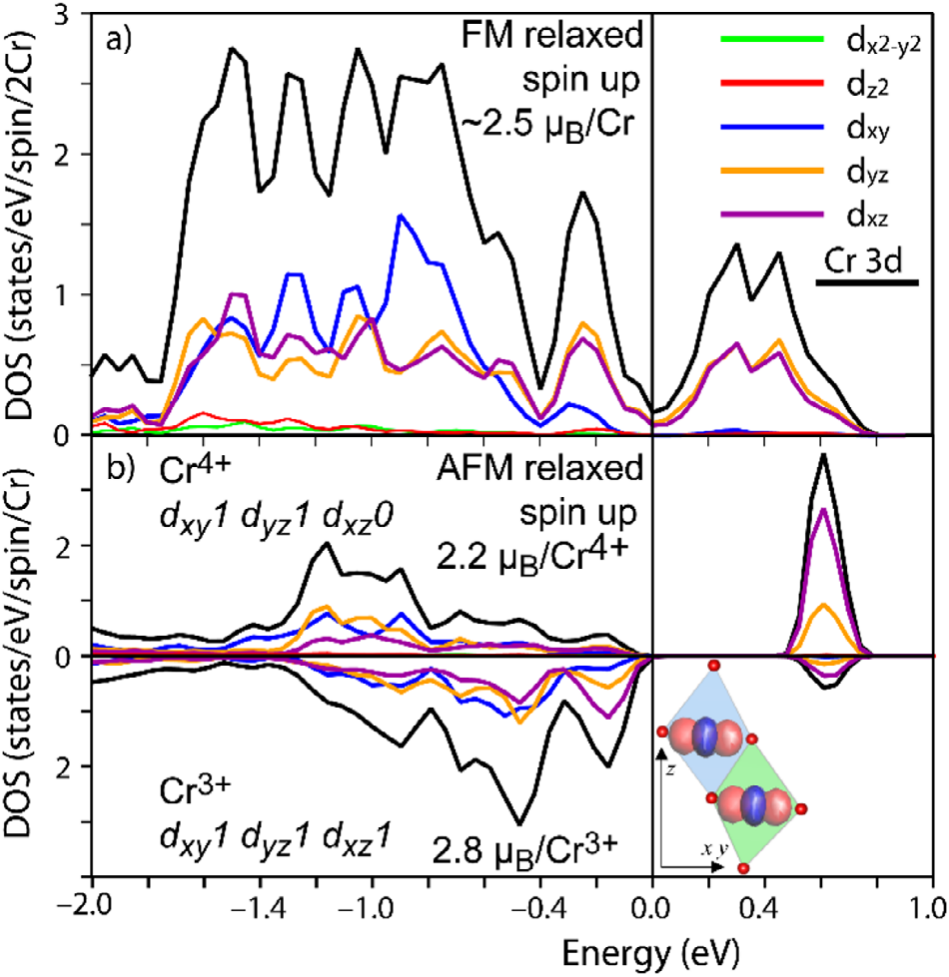
Partial density of states of K_0.75_Li_2_Cr_6_O_12_. a) Cr majority spins for FM relaxed structure. b) Cr^3+^ and Cr^4+^ majority spins AFM relaxed structure. The inset shows the selected local basis.

The calculated orbital‐decomposed partial DOS for both FM and AFM models are shown in **Figure** **5**, with the local *z*‐axis directed toward the oxygens involved in the edge‐shared connecting double chains, see inset in Figure 5b. In the mixed valence FM state, the electronic structure is that of a half‐metal, with almost a pseudogap at the Fermi level, see full DOS in Supporting Information. In the Cr *t*_2g_ majority‐spin band, the localized *d*_xy_ and itinerant *d*_yz_ and *d*_xz_ orbitals realize the FM state via an oxygen ligand‐hole double‐exchange mechanism, similar to K_2_Cr_8_O_16_ and CrO_2_.^[^ ^29^, ^30^, ^31^ ^]^ Again, our experimental observations differ from this scenario. On the contrary, the AFM partial DOS shows a narrow bandgap of ≈ 0.4 eV for almost unoccupied *d*_xz_ for Cr^4+^. *J*_3_ direct exchanges involve *d*_xy_–*d*_xy_ overlap, displaying a higher density for Cr^3+^ columns and therefore with stronger AFM interactions. However, the FM *J*_1_–*J*_2_ (Cr^3+^–Cr^4+^) origin is still elusive; it could originate via double‐exchange but without metallic behavior. Nevertheless, Goodenough–Kanamori–Andersson rules do predict FM interactions for 90° *d*_2_–*d*_3_ exchanges.^[^ ^32^, ^33^ ^]^

## 3 Discussion and Conclusion

The magnetic structure of K_0.75_Li_2_Cr_6_O_12_ can be understood in light of the exchange interactions in plane in Figure 4c; they describe a honeycomb lattice with *J*_1_ and *J*_2_ exchanges intra‐ and inter‐ zig‐zag ladders, respectively. Each ladder can be considered as a commensurate helical ordered structure with a 45° angle in between spins, see Figure 4d. The classical ground state of a single helix shows a rotational angle φ = arccos |*J*|/4*J*_3_.^[^ ^34^ ^]^ A generalized treatment, where a honeycomb lattice of interacting zig‐zag ladders is included, has also been tackled theoretically,^[^ ^35^ ^]^ explaining, for instance, the incommensurate spin helical structure in β‐CaCr_2_O_4_ with Cr^3+[^ ^22^ ^]^ or SrRE_2_O_4_.^[^ ^36^ ^]^ From these models, one can see that the necessary ingredient to induce frustration and to obtain a helical spin order, not just a trivial FM or AFM ground state, is that J_3_ > 0, that is, the exchange along the ladder legs must be antiferromagnetic and thus supporting our DFT + U AFM relaxed model discussed above. Moreover, the predicted rotational angle for K_0.75_Li_2_Cr_6_O_12_ is φ = arccos [(|*J*_1_|+2|*J*_2_|)/6*J*_3_] ≈ 50°, which is remarkably close to the observed 45° from PND. Furthermore, the calculated FM interactions in plane are clearly materialized experimentally, as shown in Figure 4c, from the 18 spins contained in this cut (it considers only ¼ of the magnetic cell along *c*_m_), 9 are oriented toward the same direction (↘) and the other 6 (→) + 3 (↓) can only increase the total magnitude in that specific cut, i.e. a FM layer. This is finally cancelled out due to the commensurate helical ordering along *c*_m_, that is, the ¼ *k*_z_ component in the propagation vector. Furthermore, the temperature evolution of the crystal structure shows an expansion in the *ab*‐plane (compatible with a Coulomb repulsion effect, i.e. FM interactions) and a contraction along the *c* direction (compatible with the AFM ordering along the chains).

As evidenced above, the decisive factor in our DFT + U calculations appears to be the contraction along the *c* parameter, changing *J*_3_ from FM to AFM. The AFM helical spin structure in the K_0.75_Li_2_Cr_6_O_12_ zig‐zag triangular ladders contrasts with the FM order observed in K_2_Cr_8_O_18_ and NaCr_2_O_4_, together with a higher “compression” along the *c*‐axis for the former (*c* = 2.86 Å) compared to the latter (*c* = 2.93–2.92 Å). Additionally, the role of this compression is highlighted in K_2_Cr_8_O_16_, where only 2.1 GPa is required to change it from a FM to an AFM behavior.^[^ ^37^, ^38^ ^]^ The reason for the abnormally short *c* parameter in K_0.75_Li_2_Cr_6_O_12_, which is built on a similar double‐rutile chain motif, remains unclear. However, the existence of fully occupied Li^+^ channels could enforce such a contraction to maintain the LiO_6_ prismatic coordination. In contrast, the K^+^ occupancy is relaxed, as hinted by the BVEL analysis and the preceding discussion. Furthermore, a comparison of the *c*‐axes on the vanadium hollandites K_2_V_8_O_16_, Tl_1.74_V_8_O_16_, Pb_1.36_V_8.35_O_16.7_, and Bi_1.7_V_8_O_16_ (2.916(2) Å, 2.899(7) Å, 2.903(1) Å, and 2.914(1) Å, respectively^[^ ^39^, ^40^, ^41^ ^]^) shows that changing the cation in the large channel does not drastically affect the cell. Thus, in K_0.75_Li_2_Cr_6_O_12_, the FM correlated paramagnet → CO AFM transition is reinforced via magnetostriction at *T*_N_ as a consequence of the pre‐existing contraction along the *c*‐axis.

Finally, the CO model used in the PND refinement at 10 K is primarily reflected in the magnetic moments, which show a clear contrast between Cr^4+^ and Cr^3+^ with 1.37(4) and 2.88(4) µ_B_, respectively. Consequently, we have reduced the symmetry to *P‐6* in the structural model to allow for the distinction of two Cr sites. Using an interpolation method,^[^ ^42^ ^]^ the BVS at 10 K (PND) resulted in values of +3.72/+3.63, which indicates no long‐range charge‐ordering. This is not surprising, as several transition metal oxides show minimal CO; for instance, in TbBa[Mn^3+^Mn^4+^]O_6_ and (LaSr_2_)Mn^3+^Mn^4+^O_7_ have BVS values of 3.45 vs. 3.67 and 3.67 vs. 3.87 for Mn^3+^ and Mn^4+^, respectively.^[^ ^38^ ^]^ This is probably due to the K^+^ intrinsic disorder, since TEM reveals no correlation between different channels. However, the spin ordering is consistent with CO within the double‐rutile chains, albeit without correlation between them, as shown in Figure 4d.

In conclusion, we have discovered the new oxide K_0.75_Li_2_Cr_6_O_12_, which stabilizes a rare mixed‐valence chromium via high‐pressure high‐temperature synthesis. By combining X‐ray and neutron diffraction, electron microscopy, and total scattering techniques, we found that K_0.75_Li_2_Cr_6_O_12_ has a different local structure that contrasts from its average structure. DFT + U calculations revealed the critical role of the compressed *c* parameter, possibly reinforced by the LiO_6_ ideal coordination, which dictates the FM or AFM behavior and suggests a FM‐correlated paramagnet → CO AFM transition. K_0.75_Li_2_Cr_6_O_12_ orders antiferromagnetically at 75 K with a *k* = [⅓ ⅓ ¼] propagation vector. The magnetic structure consists of Cr^3+^–Cr^4+^ zig‐zag ladders within a honeycomb lattice, forming a single‐angle commensurate‐ spiral spin structure with a 45° rotation. However, the lack of correlation in K^+^ positions between different channels distorts the Cr─O nearest bonds, ultimately suppressing the long‐range charge order that, at a minimum, should prevail at the columnar level of the double‐rutile chains. The mechanism described here also unravels the FM – AFM transition in K_2_Cr_8_O_16_ hollandite under pressure and points toward a more general behavior in mixed‐valence chromium oxides.

## Conflict of Interest

The authors declare no conflict of interest.

## Supporting information



Supporting Information

Supporting Information

## Data Availability

The data that support the findings of this study are available from the corresponding author upon reasonable request.
