# Supplementary material for: Hidden Charge‐Order in the Mixed‐Valent K0.75Li2Cr6O12 High‐Pressure Oxide
Source: Adv Sci (Weinh). 2025 Dec 5;13(11):e18064. doi: 10.1002/advs.202518064 (PMC12931162; doi:10.1002/advs.202518064)
Supplement: Supplementary file 1 — Supporting Information [file ADVS-13-e18064-s002.docx]

Supplementary Information for:

**Hidden charge-order in the mixed-valent K_0.75_Li_2_Cr_6_O_12_ high-pressure oxide.**

Angel M. Arévalo-López,*^[a]^ Clemens Ritter,^[b]^ Marielle Huvé,^[a]^ and Olivier Mentré^[a]^

[a] A. M. Arévalo-López, M. Huvé, O. Mentré
Unité de Catalyse et Chimie du Solide (UCCS),

Université de Lille, Centrale Lille/ENSCL. 59000 Lille, France.
angel.arevalo-lopez@univ-lille.fr

[b] C. Ritter
Institut Laue-Langevin
71 Avenue des Martyrs, Cedex, Grenoble 32042, France

**Experimental Methods:**

Polycrystalline K_0.75_Li_2_Cr_6_O_12_ was obtained at 12 GPa and 1373 K for 30 minutes using a 1000 Tons hydraulic press (Savoisienne de Verins Hydrauliques) with a multi-anvil Walker-type module (Voggenreiter). Aiming for KLi_2_Cr_6_O_12_, stoichiometric amounts of LiCrO_2_, K_2_Cr_2_O_7_ (Pierron S.A., 99%) and Cr_2_O_3_ (Prolabo, 99.9%) were placed in a Pt capsule and into the press. LiCrO_2_ was prepared from a 1.05 : 1 Li_2_CO_3_ (Sigma Aldrich, 99.9%): Cr_2_O_3_ mixture in an alumina crucible in air at 800 °C for 24 h.

Preliminary laboratory X-ray powder diffraction patterns were collected using Cu-Ka radiation on a Bruker D8 Advance diffractometer in the 5° < 2θ < 90° range.

Electron diffraction was performed on a FEI Technai G2-20 twin TEM microscope equipped with an EDX spectrometer. A drop of an alcohol suspension with crushed powder was deposited on a carbon-supported copper grid and then let for evaporation under ambient conditions before being inserted into the microscope.

High-resolution synchrotron X-ray diffraction and total scattering measurements were performed at beamline ID31 at the European Synchrotron Radiation Facility (ESRF). The sample powder was loaded in a 1 mm capillary. K_0.75_Li_2_Cr_6_O_12_ was measured in transmission geometry with an incident X-ray energy of 75.051 keV (λ = 0.16520 Å). PDF data were processed in an automated way using PDFgetX3 [^[[1]](#endnote-1)^, ^[[2]](#endnote-2)^, ^[[3]](#endnote-3)^]. Then the data were reprocessed using a Lorch modification function [^[[4]](#endnote-4)^] to suppress termination effects and contributions from high frequency noise.

High-resolution neutron powder diffraction (NPD) data were collected in a combination of several high-pressure experiment runs (63 mg in total) in the D20 diffractometer at the ILL European neutron facility in Grenoble. The NPD 2θ scans at 10 K and at 100 K in the high-resolution mode at 90° take-off and with 1.54 A wavelength neutrons used to create the difference data file and the T-dependent ramp which were measured using the same high resolution take-off angle but with 2.42 A neutrons. The ramp speed was 0.1 K every 36 secs giving a delta T of 1.67 K between adjacent datasets. Rietveld refinements were performed using Fullprof Suite.[^[[5]](#endnote-5)^]

Magnetisation, heat capacity and resistivity measurements were obtained from experiments in a Quantum Design PPMS 9T Dynacool. Magnetic susceptibilities were measured from 2 K to 300 K under a m_0_H = 0.1 T magnetic field using zero-(ZFC) and field-cooling (FC) procedures. Magnetisation versus field isotherms were measured between 9 and 9 T. Van-der-Paw method was used for resistivity measurements.

DFT+U calculations were performed with the general gradient approximation (GGA) using the Perdew–Burke–Ernzerhof (PBE) approach in the Vienna ab initio simulation package (VASP).[^[[6]](#endnote-6)^] Extra on-site electron–electron Coulomb interactions were also considered with the GGA+U approach implemented in the code. Crystal structures were optimized by minimizing the total energy. The lattices and atomic positions were relaxed starting from the refined structural model but removing one of the K^+^ positions to avoid disorder as explained in the main text, within a convergence force criterion of 0.03 eV Å^-1^. The cut-off energy for the plane-wave-basis is 400 eV, and the total energy of the system was converged with respect to the plane-wave cut-off energy and reciprocal space samplings. The free-energy convergence criterion was set to 10^-10^ eV using a G-centered 8 x 8 x 8 Monkhorst-Pack k-point mesh.

**Figure S1.** a) EDX spectra of the K_2_Cr_2_O_7_ standard with a 1:1 K:Cr ratio chemical ratio. b) EDX spectra of K_0.75_Li_2_Cr_6_O_12_ sample resulting in a 1:8 K:Cr ratio and therefore the 0.75 K occupancy. This was collected in more than 15 crystals in different areas resulting in the same K:Cr ratio.

a)
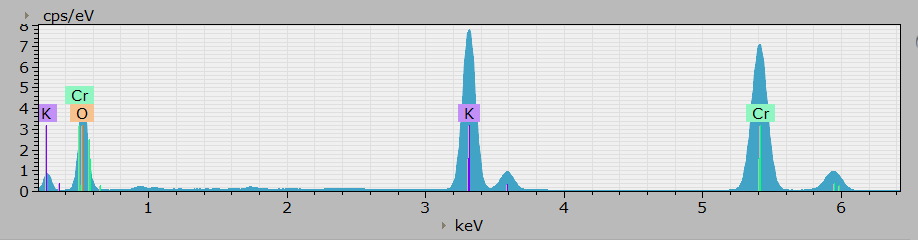


b)
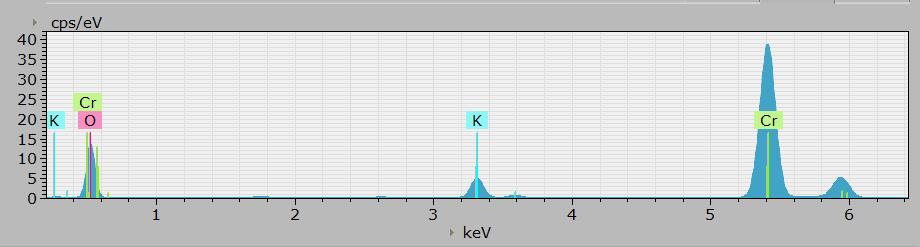


**Figure S2.** DFT + U calculation of K_0.75_Li_2_Cr_6_O_12_ in a 2*a* x *b* x 4*c* supercell, in order to probe the influence of the K+/Vc distribution and the communication between the different channels. a) Initial configuration selecting two different vacancy positions. b) Relaxed structure showing the redistribution of the K^+^ along the channel in order to minimize the vacancy influence. The oxygen position closer to the K^+^ channel is the one that moves the most. CIF file is attached as a separate file


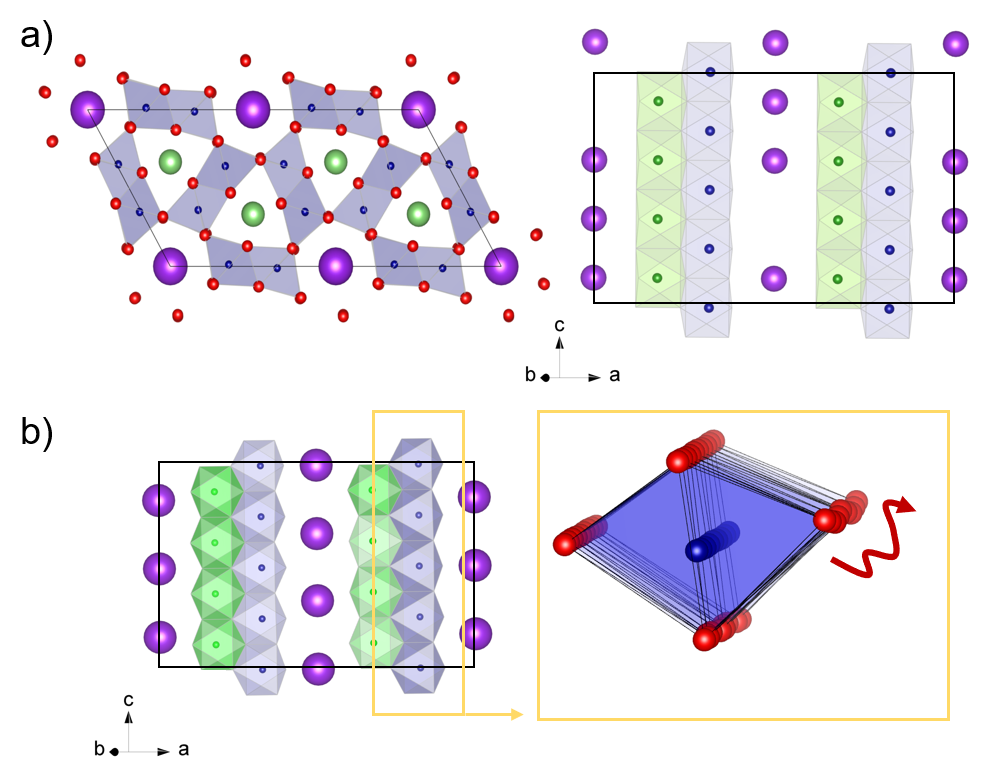


**Figure S3.** PDF fit for K_0.75_Li_2_Cr_6_O_12_ in a *a* x *b* x 4*c* supercell at short distances. BVS calculations show clear Cr^3+^ and Cr^4+^ columnar order but half of the CrO_6_ octahedra are still in a mixed valent state.


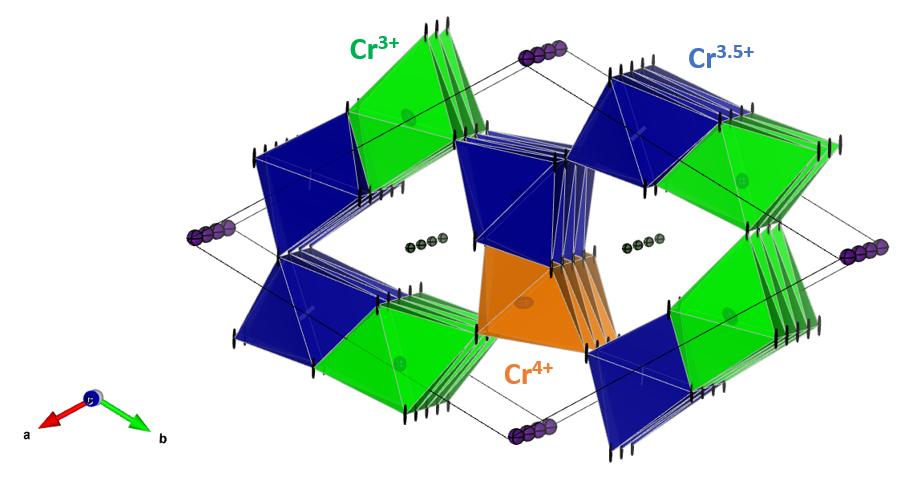


**Figure S4.** Arrhenius plot of resistivity with an activation energy of 0.17(2) eV. The fit was performed in the 200 K < T < 300 K temperature range.


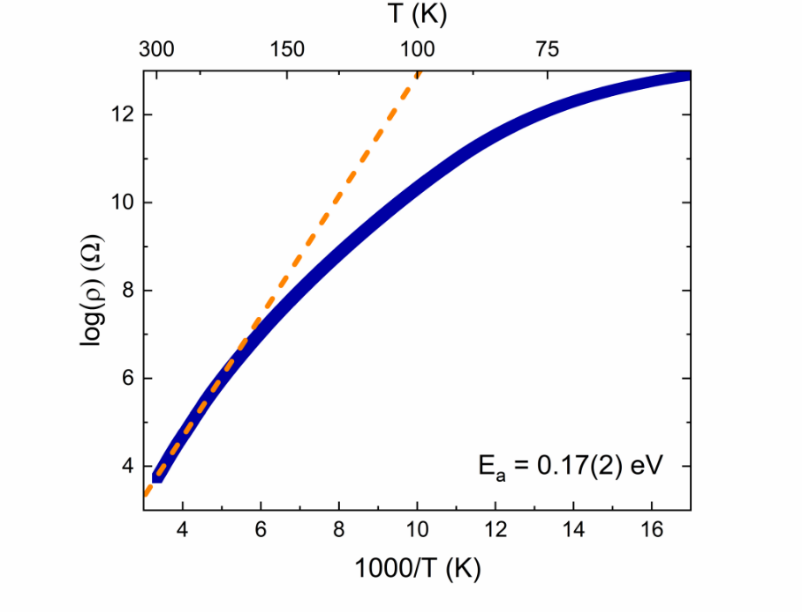


**Figure S5.** Resistivity measurements under 0 and 9 T magnetic field, demonstrating a negligible magnetoresistance.


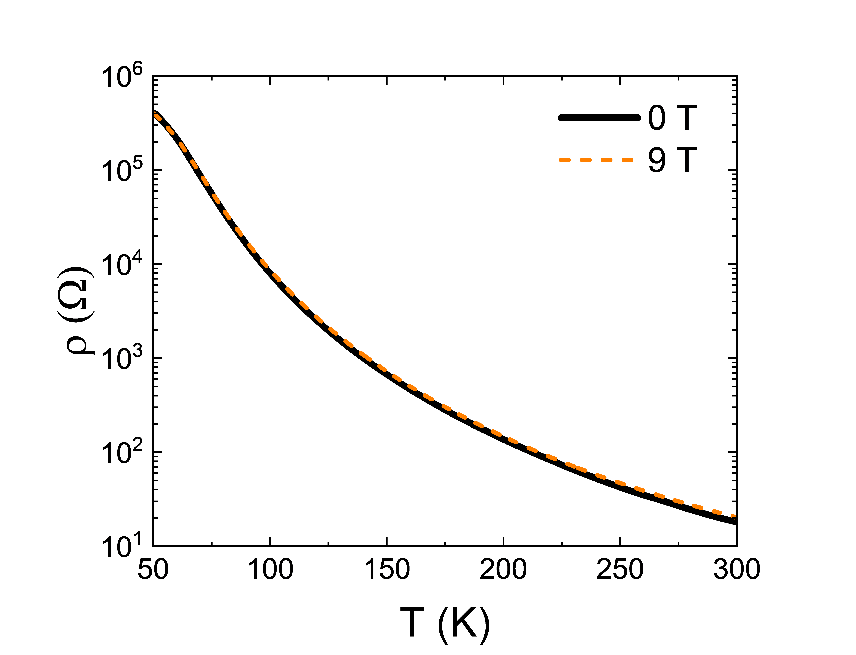


**Figure S6**. Magnetic contribution to the neutron diffraction obtained from 100 K subtraction to the 10 K data. The fit was performed with two different propagation vectors *k_1_* = [1/3 1/3 ¼] and *k_2_* = [0 0 ¼] but later realized that *k_2_* is a second order parameter of *k_1_* and a better description is via the magnetic space group as detailed in the main text. The main reflection arises from the 45° spiral ordering along *c*.


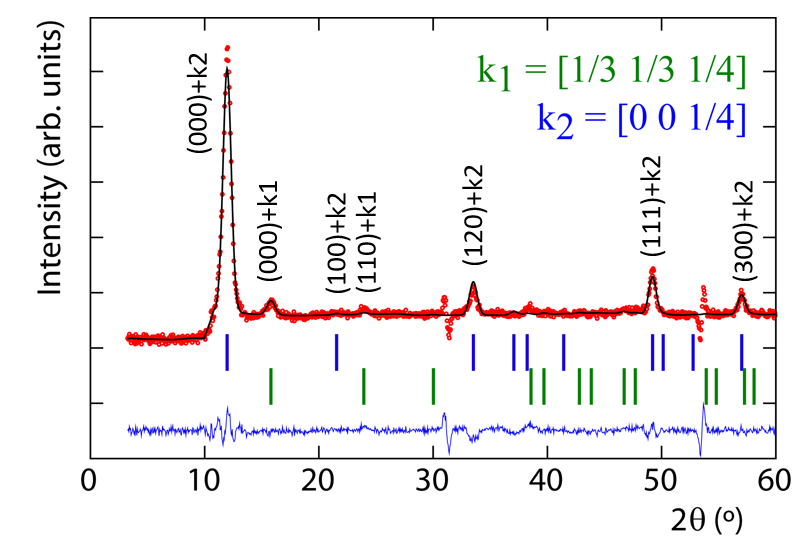


**Figure S7**. Crystal and magnetic structure at 10 K. The fit to the structure was performed with *P-6* space group which allows for two independent chromium sites, see Supplementary Tables below. Secondary phases LiCrO_2_ (4.6 wt%) and Cr_2_O_3_ (6 wt%), along with the sample holder V and the magnetic structure where also included in the refinement as 2^nd^, 3^rd^, 4^th^ and 5^th^ phases.


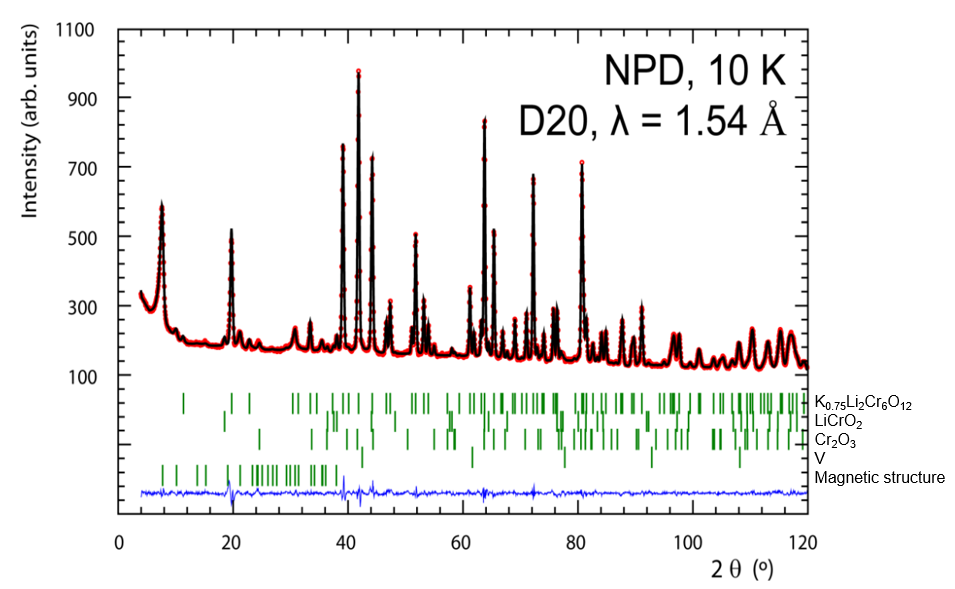


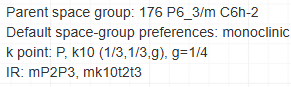

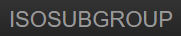

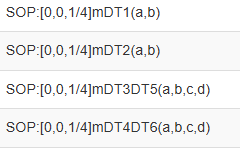
**Figure S8.** Snapshots of the input for the ISOSUBGROUP program within the ISODISTORT suite of packages.[^[[7]](#endnote-7)^,^[[8]](#endnote-8)^] It shows that *k_2_* = [0 0 ¼] is a second order parameter of *k_1_* = [1/3 1/3 ¼].

**Figure S9**. DFT+U calculation from the refined model (a) with a FM (b) and an AFM (c) configuration that implies charge order of the chromium in columns.


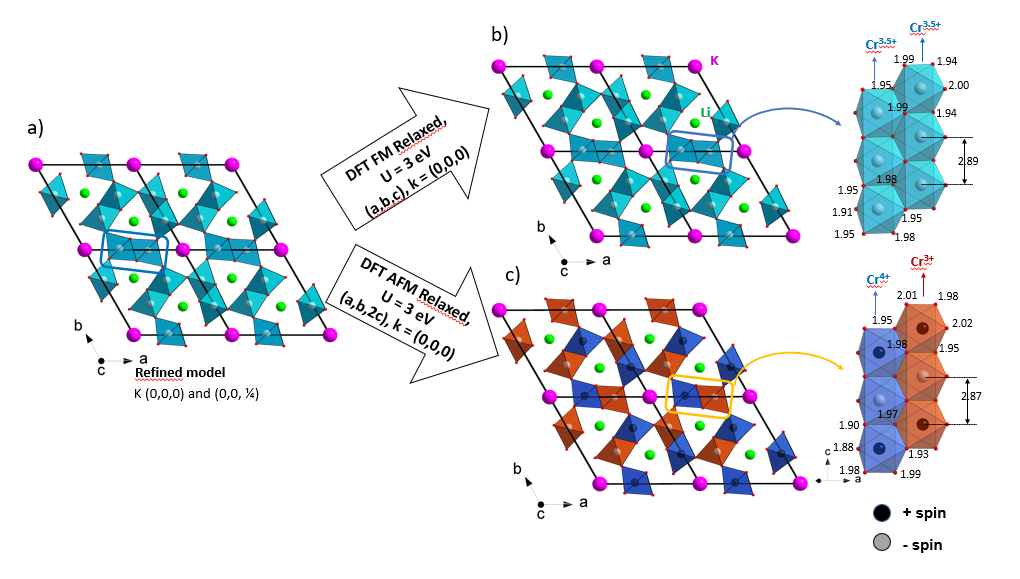


**Figure S10**. Comparison between the cell parameters thermal behavior around *T_N_* and the DFT + U cell parameters relaxed in the FM and the AFM configurations from Figure S9.

**
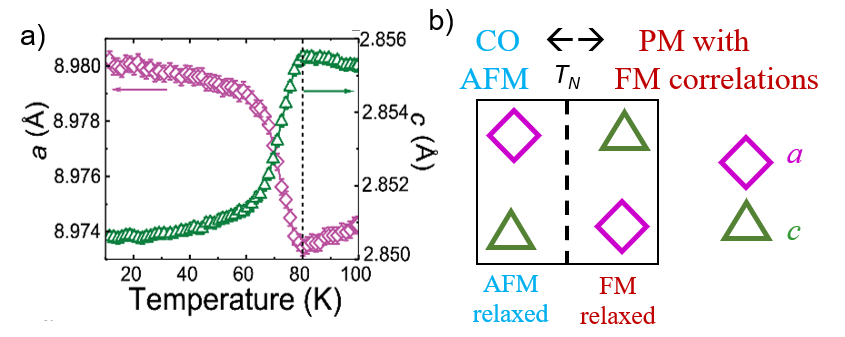
**

**Figure S11**. Density of states of the FM relaxed model showing the half-metal behavior similar to CrO_2_ and K_2_Cr_8_O_16_ hollandite. Total in black, chromium in blue and oxygen in red.


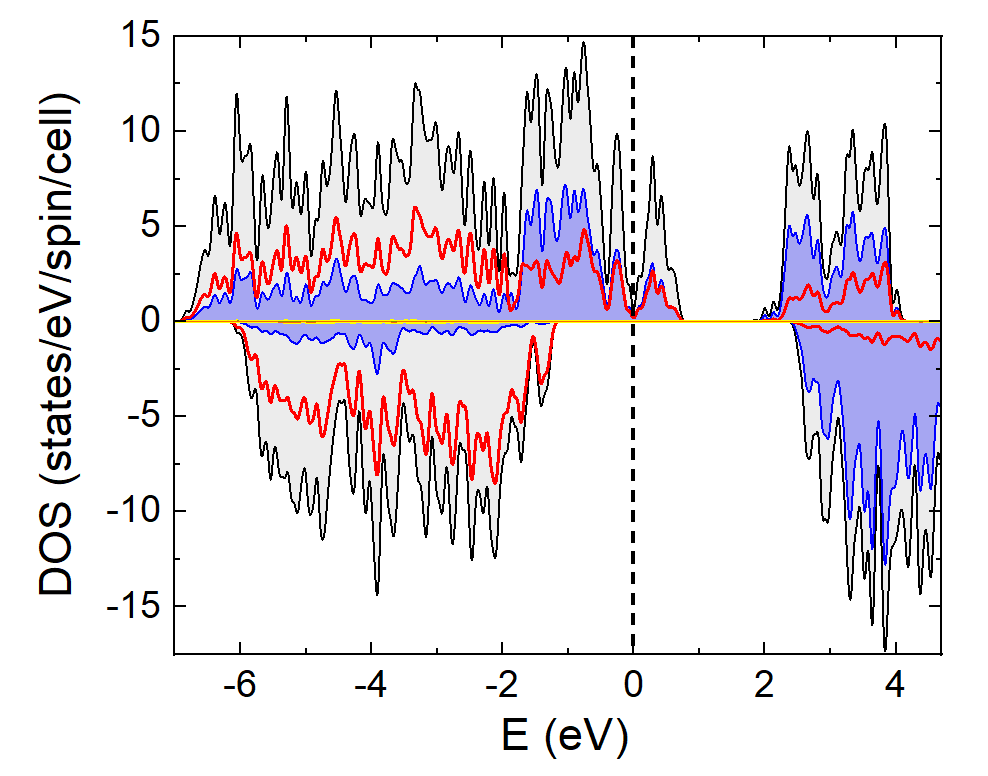


**Table S1.** Atomic coordinates and selected bond distances of K_0.75_Li_2_Cr_6_O_12_ from the Rietveld fit of SXRD at 300 K data S.G. *P6_3_/m*, *a* = 9.010982(2) Å, *c* = 2.86296(1) Å. *R*_exp_ = 1.90 *R_f_* = 2.56, *R*_B_ = 3.73.

| **Site** | **x** | **y** | **z** | **BVS** |
| --- | --- | --- | --- | --- |
| K (2a)^a^ | 0 | 0 | 0.36291(5) | 1.378(6) |
| Li(2c) | 2/3 | 1/3 | ¾ | 0.971(2) |
| Cr(6h) | 0.36048(6) | 0.34832(8) | ¾ | 3.488(10) |
| O1(6h) | 0.5348(3) | 0.4020(3) | ¼ | 1.963(6) |
| O2(6h) | 0.6840(3) | 0.8865(3) | ¼ | 2.108(7) |
| **bond** | **length (Å)** | **bond** | **length (Å)** |  |
| Cr-O1 x2 | 1.998(1) | K-O2 x3 | 2.519(3) |  |
| Cr-O1 x1 | 1.957(3) | K-O2 x3 | 2.773(3) |  |
| Cr-O2 x2 | 1.935(3) | K-O2 x3 | 3.053(3) |  |
| Cr-O2 x1 | 1.947(3) | Li-O1 x6 | 2.140(3) |  |

^a^ refined to 0.182(1) (4e site, *i.e.* 0.728 occupied)

**Table S2.** Anisotropic displacement parameters for Table S1 (x10^4^).

| **Site** | **B_11** | **B_22** | **B_33** | **B_12** | **B_13** | **B_23** |
| --- | --- | --- | --- | --- | --- | --- |
| K (2a) | 60(30) | 60(30) | 50(12) | 30(30) | 0 | 0 |
| Li(2c) | 10(5) | 10(5) | 370(7) | 0(5) | 0 | 0 |
| Cr(6h) | 95(4) | 114(4) | 76(3) | 48(3) | 0 | 0 |
| O1(6h) | 50(13) | 50(13) | 81(14) | -4(10) | 0 | 0 |
| O2(6h) | 73(13) | 96(13) | 57(11) | 50(12) | 0 | 0 |

**Table S3.** Atomic coordinates and selected bond distances of K_0.75_Li_2_Cr_6_O_12_ from the Rietveld fit of PND at 200 K data S.G. *P6_3_/m*, *a* = 8.98120(13) Å, *c* = 2.85482(5) Å. *R*_exp_ = 0.98 *R_f_* = 2.04, *R*_B_ = 2.81.

| **Site** | **x** | **y** | **z** | **BVS** |
| --- | --- | --- | --- | --- |
| K (2a)^a^ | 0 | 0 | 0.351(3) | 1.378(6) |
| Li(2c) | 2/3 | 1/3 | ¾ | 0.971(2) |
| Cr(6h) | 0.36039(19) | 0.34803(24) | ¾ | 3.488(10) |
| O1(6h) | 0.53157(17) | 0.40284(15) | ¼ | 1.963(6) |
| O2(6h) | 0.68416(13) | 0.88650(13) | ¼ | 2.108(7) |
| **bond** | **length (Å)** | **bond** | **length (Å)** |  |
| Cr-O1 x2 | 1.9714(17) | K-O2 x3 | 2.5054(16) |  |
| Cr-O1 x1 | 1.944(3) | K-O2 x3 | 2.737(4) |  |
| Cr-O2 x2 | 1.9303(17) | K-O2 x3 | 3.023(5) |  |
| Cr-O2 x1 | 1.937(4) | Li-O1 x6 | 2.1580(15) |  |

^a^ fixed to 0.1875 (4e site, *i.e.* 0.75 occupied), all the other occupancies refined to nominal values within error and kept fixed. Isotropic thermal factors constrained together and refined to K^+^ Biso = 0.78(24) Å^2^.

**Table S4.** Anisotropic displacement parameters for Table S3 (x10^4^).

| **Site** | **U_11** | **U_22** | **U_33** | **U_12** | **U_13** | **U_23** |
| --- | --- | --- | --- | --- | --- | --- |
| Li(2c) | 300(16) | 300(50) | 400(40) | 150(5) | 0 | 0 |
| Cr(6h) | 52(4) | 55(4) | 19(3) | 29(3) | 0 | 0 |
| O1(6h) | 61(6) | 68(7) | 47(5) | 34(5) | 0 | 0 |
| O2(6h) | 89(6) | 71(6) | 41(5) | 43(6) | 0 | 0 |

**Table S5.** Atomic coordinates and selected bond distances of K_0.75_Li_2_Cr_6_O_12_ from the Rietveld fit of PND at 10 K data S.G. *P-6*, *a* = 8.97975(16) Å, *c* = 2.85105(5) Å. *R*_exp_ = 0.99 *R_f_* = 1.70, *R*_B_ = 2.27.

| **Site** | **x** | **y** | **z** | **BVS** |
| --- | --- | --- | --- | --- |
| K (2g)^a^ | 0 | 0 | 0.129(9) | 1.917(6) |
| Li-a (1f) | 2/3 | 1/3 | ½ | 0.982(7) |
| Li-b (1c) | 1/3 | 2/3 | 0 | 0.859(6) |
| Cr-a (3k)^b^ | 0.3598(12) | 0.3549(13) | ½ | 3.72(4) |
| Cr-b (3j)^b^ | 0.6382(13) | 0.6577(14) | 0 | 3.63(6) |
| O1-a (3j) | 0.5302(9) | 0.3970(9) | 0 | 1.85(3) |
| O1-b (3k) | 0.4683(9) | 0.5915(8) | ½ | 2.37(4) |
| O2-a (3j) | 0.6821(14) | 0.8850(11) | 0 | 2.29(5) |
| O2-b (3k) | 0.3145(14) | 0.1109(11) | ½ | 2.09(4) |
| **bond** | **length (Å)** | **bond** | **length (Å)** |  |
| Cra-O1a x2 | 1.984(10) | Crb-O1b x2 | 1.952(10) |  |
| Cra-O1b x1 | 1.842(14) | Crb-O1a x1 | 2.037(14) |  |
| Cra-O2b x1 | 2.018(18) | Crb-O2a x1 | 1.877(18) |  |
| Cra-O2a x2 | 1.912(12) | Crb-O2b x2 | 1.939(13) |  |
| K-O2a x3 | 2.531(13) | Lia-O1a x6 | 2.136(18) |  |
| K-O2b x3 | 2.697(12) | Lib-O1b x6 | 2.185(8) |  |
| K-O2b x3 | 3.061(11) |  |  |  |

^a^ fixed to 0.375 (2g site, *i.e.* 0.75 occupied). ^b^ Biso = 0.41(5) constraint to be equal.

**Table S6.** Anisotropic displacement parameters for Table S5 (x10^4^).The thermal factors were constrained to be the same for O1 a and b; O2 a and b; Cr a and b and Li a and b.

| **Site** | **B_11** | **B_22** | **B_33** | **B_12** | **B_13** | **B_23** |
| --- | --- | --- | --- | --- | --- | --- |
| K | 54(19) | 54(19) | 85(8) | 10(3) | 0 | 0 |
| Li (a & b) | 82(18) | 82(18) | 400(40) | 0(5) | 0 | 0 |
| O1 (a & b) | 28(3) | 16(3) | 83(15) | 10(3) | 0 | 0 |
| O2 (a & b) | 41(4) | 30(3) | 88(14) | 25(3) | 0 | 0 |

**Table S7.** Atomic coordinates and selected bond distances of K_0.75_Li_2_Cr_6_O_12_ from the PDF fit of SXRD at 300 K data using *P6_3_/m* constraints, *a* = 9.007(2) Å, *c* = 2.8618(9) Å. χ^2^ =0.0077, *R*_w_ = 0.1584.

| **Site** | **x** | **y** | **z** | **BVS** |
| --- | --- | --- | --- | --- |
| K (2a)^a^ | 0 | 0 | 0.225(7) | 1.378(6) |
| Li(2c) | 2/3 | 1/3 | ¾ | 0.971(2) |
| Cr(6h) | 0.3601(6) | 0.3482(6) | ¾ | 3.488(10) |
| O1(6h) | 0.532(3) | 0.403(3) | ¼ | 1.963(6) |
| O2(6h) | 0.683(4) | 0.885(4) | ¼ | 2.108(7) |
| **bond** | **length (Å)** | **bond** | **length (Å)** |  |
| Cr-O1 x2 | 1.982(65) | K-O2 x3 | 2.505(15) |  |
| Cr-O1 x1 | 1.942(56) | K-O2 x3 | 2.849(12) |  |
| Cr-O2 x2 | 1.935(50) | K-O2 x3 | 2.920(30) |  |
| Cr-O2 x1 | 1.939(87) | Li-O1 x6 | 2.163(47) |  |

^a^ refined to 0.182(1) (4e site, *i.e.* 0.728 occupied)

**Table S8.** Anisotropic displacement parameters for Table S7 (x10^4^).

| **Site** | **B_11** | **B_22** | **B_33** | **B_12** | **B_13** | **B_23** |
| --- | --- | --- | --- | --- | --- | --- |
| K (2a) | 24(66) | 24(66) | 1284(340) | 12(33) | 0 | 0 |
| Li(2c) | 51(92) | 51(92) | 285(57) | 25(46) | 0 | 0 |
| Cr(6h) | 27(8) | 35(8) | 28(6) | 11(7) | 0 | 0 |
| O1(6h) | 97(47) | 101(44) | 69(47) | 45(40) | 0 | 0 |
| O2(6h) | 181(60) | 139(59) | 53(50) | 12(6) | 0 | 0 |

**Table S9.** Atomic coordinates of KLi_2_Cr_6_O_12_ from DFT in the FM configuration. S.G. *P1*, *a* = 8.98747 Å, *c* = 2.89359 Å. See main text. CIF attached as separate supplementary file.

| **Site** | **x** | **y** | **z** | **BVS** |
| --- | --- | --- | --- | --- |
| K | 0 | 0 | 0 | 2.011 |
| Li1 | 2/3 | 1/3 | 0 | 1.008 |
| Li2 | 1/3 | 2/3 | ½ | 0.971 |
| Cr1 | 0.65580 | 0.64158 | 0 | 3.544 |
| Cr2 | 0.35842 | 0.01422 | 0 | 3.544 |
| Cr3 | 0.98578 | 0.34420 | 0 | 3.544 |
| Cr4 | 0.35195 | 0.36336 | ½ | 3.426 |
| Cr5 | 0.63664 | 0.98859 | ½ | 3.426 |
| Cr6 | 0.01141 | 0.64805 | ½ | 3.426 |
| O1 | 0.46545 | 0.87095 | 0 | 1.972 |
| O2 | 0.12905 | 0.59450 | 0 | 1.972 |
| O3 | 0.40550 | 0.53455 | 0 | 1.972 |
| O4 | 0.52836 | 0.13065 | ½ | 1.995 |
| O5 | 0.86935 | 0.39771 | ½ | 1.995 |
| O6 | 0.60229 | 0.47164 | ½ | 1.995 |
| O7 | 0.32223 | 0.20696 | 0 | 2.250 |
| O8 | 0.79304 | 0.11527 | 0 | 2.250 |
| O9 | 0.88473 | 0.67777 | 0 | 2.250 |
| O10 | 0.69837 | 0.80367 | ½ | 2.083 |
| O11 | 0.19633 | 0.89470 | ½ | 2.083 |
| O12 | 0.10530 | 0.30163 | ½ | 2.083 |

**Table S10.** Atomic coordinates of KLi_2_Cr_6_O_12_ from DFT in the AFM configuration. S.G. *P1*, *a* = 9.02274 Å, 2*c* = 5.75163 Å. See main text. CIF attached as separate supplementary file.

| **Site** | **x** | **y** | **z** | **BVS** | **Site** | **x** | **y** | **z** | **BVS** |  |  |  |
| --- | --- | --- | --- | --- | --- | --- | --- | --- | --- | --- | --- | --- |
| K1 | 0 | 0 | -0.0057 | 0.9855 | O4 | 0.12916 | 0.58947 | 0.49813 | 1.980 |  | | |
| K2 | 0 | 0 | 0.4943 | 0.9855 | O5 | 0.41033 | 0.53949 | -0.00187 | 1.980 |  |  |  |
| Li1 | 2/3 | 1/3 | 0.00368 | 1.031 | O6 | 0.41033 | 0.53949 | 0.49813 | 1.980 |  |  |  |
| Li2 | 2/3 | 1/3 | 0.50368 | 1.031 | O7 | 0.52914 | 0.13233 | 0.25411 | 1.998 |  |  |  |
| Li3 | 1/3 | 2/3 | 0.25767 | 0.992 | O8 | 0.52914 | 0.13233 | 0.75411 | 1.998 |  |  |  |
| Li4 | 1/3 | 2/3 | 0.75767 | 0.992 | O9 | 0.86767 | 0.39680 | 0.25411 | 1.998 |  |  |  |
| Cr1 | 0.65647 | 0.64107 | 0.00642 | 3.667 | O10 | 0.86767 | 0.39680 | 0.75411 | 1.998 |  |  |  |
| Cr2 | 0.65647 | 0.64107 | 0.50642 | 3.667 | O11 | 0.60320 | 0.47086 | 0.25411 | 1.998 |  |  |  |
| Cr3 | 0.35893 | 0.01539 | 0.00642 | 3.667 | O12 | 0.60320 | 0.47086 | 0.75411 | 1.998 |  |  |  |
| Cr4 | 0.35893 | 0.01539 | 0.50642 | 3.667 | O13 | 0.32135 | 0.20266 | -0.00195 | 2.222 |  |  |  |
| Cr5 | 0.98461 | 0.34353 | 0.00642 | 3.667 | O14 | 0.32135 | 0.20266 | 0.49805 | 2.222 |  |  |  |
| Cr6 | 0.98461 | 0.34353 | 0.50642 | 3.667 | O15 | 0.79734 | 0.11869 | -0.00195 | 2.222 |  | |  |
| Cr7 | 0.35348 | 0.36513 | 0.24508 | 3.268 | O16 | 0.79734 | 0.11869 | 0.49805 | 2.222 |  | |  |
| Cr8 | 0.35348 | 0.36513 | 0.74508 | 3.268 | O17 | 0.88131 | 0.67865 | -0.00195 | 2.222 |  | |  |
| Cr9 | 0.63487 | 0.98835 | 0.24508 | 3.268 | O18 | 0.88131 | 0.67865 | 0.49805 | 2.222 |  | |  |
| Cr10 | 0.63487 | 0.98835 | 0.74508 | 3.268 | O19 | 0.69644 | 0.80209 | 0.24632 | 2.066 |  | |  |
| Cr11 | 0.01165 | 0.64652 | 0.24508 | 3.268 | O20 | 0.69644 | 0.80209 | 0.74632 | 2.066 |  | |  |
| Cr12 | 0.01165 | 0.64652 | 0.74508 | 3.268 | O21 | 0.19791 | 0.89436 | 0.24632 | 2.066 |  | |  |
| O1 | 0.46051 | 0.87084 | -0.00187 | 1.980 | O22 | 0.19791 | 0.89436 | 0.74632 | 2.066 |  | |  |
| O2 | 0.46051 | 0.87084 | 0.49813 | 1.980 | O23 | 0.10564 | 0.30356 | 0.24632 | 2.066 |  | |  |
| O3 | 0.12916 | 0.58947 | -0.00187 | 1.980 | O24 | 0.10564 | 0.30356 | 0.74632 | 2.066 |  | |  |

1. [] P. F. Peterson, E. S. Božin, Th. Proffen, and S. J. L. Billinge. “Improved measures of quality for atomic pair distribution functions.” *J. Appl. Cryst.* **2003**, *36*, 53. [↑](#endnote-ref-1)
2. [] S. J. L. Billinge, C. L. Farrow. “Towards a robust ad-hoc data correction approach that yields reliable atomic pair distribution functions from powder diffraction data.” *J. Phys. Cond. Matt.* **2013**, *25*, 454202. [↑](#endnote-ref-2)
3. [] P. Juhas, T. Davis, C. L. Farrow, S. J. L. Billinge. “PDFgetX3: A rapid and highly automatable program for processing powder diffraction data into total scattering pair distribution functions.” *J. Appl. Cryst.* **2013**, *46*, 560. [↑](#endnote-ref-3)
4. [] E. Lorch. “Neutron diffraction by germania, silica and radiation-damaged silica glasses.” *J. Phys. C: Solid State Phys.* **1969**, *2*, 229. [↑](#endnote-ref-4)
5. [] J. Rodriguez-Carvajal, “Recent advances in magnetic structure determination by neutron powder diffraction” *Physica B,* **1993**, *192*, 55. [↑](#endnote-ref-5)
6. [] G. Kresse, J. Hafner, “Ab initio molecular Dynamic for liquid metals” *Phys. Rev. B* **1993**, *47*, 558(R). [↑](#endnote-ref-6)
7. [] H.T. Stokes, D. M. Hatch, B. J. Campbell, “ISOSUBGROUP, ISOTROPY Software Suite, iso.byu.edu” [↑](#endnote-ref-7)
8. [] H.T. Stokes, S. van Orden, B. J. Campbell, “Tool for generating isotropy subgroups of crystallographic space groups”. *J. Appl. Cryst.* **2016**, *49*, 1849. [↑](#endnote-ref-8)
